# Chronic kidney disease in lithium-treated patients, incidence and rate of decline

**DOI:** 10.1186/s40345-020-00204-2

**Published:** 2021-01-04

**Authors:** Arjan M. Van Alphen, Tessa M. Bosch, Ralph W. Kupka, Rocco Hoekstra

**Affiliations:** 1 Department of Nephrology, Maasstad Hospital, Rotterdam, The Netherlands; 2 Department of Hospital Pharmacy, Maasstad Hospital, Rotterdam, The Netherlands; 3 Dept. of Psychiatry & Amsterdam Public Health Research Institute, Amsterdam UMC, Vrije Universiteit, Amsterdam, The Netherlands; 4 Outpatient Clinic for Bipolar Disorders, Antes Centre for Mental Health Care, Rotterdam, The Netherlands

**Keywords:** Lithium, Kidney, Renal failure, Nephropathy, Chronic kidney disease

## Abstract

**Background:**

Lithium-induced nephropathy is a known long-term complication, sometimes limiting the use of lithium as mood stabilizer. The aim of this study is to establish the incidence of chronic kidney disease and the rate of decline of renal function in patients using lithium and to identify risk factors.

**Methods:**

We selected 1012 patients treated with lithium from the laboratory database of the Antes Centre for Mental Health Care spanning a period from 2000 to 2015. Serum lithium and creatinine concentrations were retrieved and eGFR was calculated using the 4-variable CKD-EPI formula. We calculated the incidence of renal insufficiency and the rate of decline. We compared patients with and without chronic kidney disease (CKD) stage 3 regarding duration of lithium exposure.

**Results:**

Incidence of chronic kidney disease was 0.012 cases per exposed patient-year. Average decline of eGFR was 1.8 ml/min/year in patients who developed chronic kidney disease stage 3. Incidence of chronic kidney disease stage 4 was only 0.0004 per patient year. No cases of end stage renal disease were found in this cohort. Odds of reaching chronic kidney disease stage 3 were increased with longer duration of lithium exposure.

**Conclusions:**

The use of lithium seems to be related to a higher incidence of chronic kidney disease. Longer duration of lithium exposure significantly increased the risk of renal failure.

## Background

After decades of use, lithium is still considered to be the gold standard therapy for bipolar disorders. Not only is lithium effective for managing acute manic or depressive episodes in bipolar disorder, it also acts as a prophylactic agent in prevention of such episodes, and has an independent anti-suicidal effect (Severus et al. 2018; Smith and Cipriani 2017). Patients using lithium salts may experience several side effects: hypothyroidism, hyperparathyroidism, nephrogenic diabetes insipidus, and decline in renal function (Gitlin 2016). The occurrence of renal failure is especially problematic in the long term. Not only is renal failure a risk factor for occurrence of cardiovascular disease, it can also make it necessary to weigh the balance between pros and cons of continuing lithium therapy.

Some uncertainty remains about the incidence of renal failure in patients treated with lithium salts. Although several reports suggest little effect of lithium on renal function, other studies estimate prevalence of chronic kidney disease (CKD) amongst lithium users between 10 and 35% (Presne et al. 2003; Lepkifker et al. 2004; Bassilios et al. 2008; Bendz et al. 2010; Aprahamian et al. 2014; Aiff et al. 2015; Tondo et al. 2017). Data about the occurrence of lithium-induced end-stage renal disease (ESRD) are sparse (Presne et al. 2003; Bendz et al. 2010; Aiff et al. 2015; Tondo et al. 2017). Nielsen et al. (2018) reviewed several large observational studies and suggested that surveillance bias might play a role. Poorer renal outcome was found in older studies while in more recent studies no end-stage renal failure was found, probably due to improved monitoring. Risk factors for renal failure seem to be higher lithium levels, duration of lithium therapy, lower initial eGFR, older age and medical comorbidity (Tondo et al. 2017; Davis et al. 2018).

It is important to more accurately define exact incidence of lithium nephropathy and contributing risk factors to better inform patients of their risk. The aim of the present study is to establish the incidence of chronic kidney disease and the course of renal function in patients treated with lithium salts.

## Methods

### Study population

The laboratory database of the Antes Centre for Mental Health Care in Rotterdam, the Netherlands, was screened for patients who used lithium. Data could be retrieved between 2000 and 2015. A total of 1751 patients were identified in whom serum lithium was measured. Patients were deemed to be on chronic lithium therapy when a minimum of 5 serum lithium measurements were available, leaving 1151 patients. Another 139 patients were excluded because eGFR was 60 ml/min or less at the initiation of lithium therapy or the start of our database in January 2000. Ultimately 1012 patients were included for analysis. Data on eGFR were available from the onset of therapy in 531 patients who started lithium after January 2000. The remaining 481 patients had initiated treatment prior to January 2000 and no data on eGFR were available between the onset of lithium therapy and the year 2000.

eGFR was calculated for each serum creatinine value, using the 4-variable CKD EPI formula. The date at which lithium was initiated or discontinued was retrieved from psychiatric records. Because we had several creatinine measurements we could calculate the average decline of the eGFR per year in the first 10 years after onset of lithium therapy. Chronic kidney disease was defined as CKD stage 3, meaning an eGFR less than 60 ml/min/1.73 m^2^. Threshold had to be met on at least two consecutive measurements spanning a 30 day period, for an individual to be considered having chronic kidney disease. The occurrence of CKD stage 3B (eGFR between 30 and 45 ml/min/1.73 m^2^) and CKD stage 4 (eGFR between 15 and 30 ml/min/1.73 m^2^) were also reported.

We calculated the incidence of CKD in the whole group. In the subgroup of patients who started lithium treatment after the year 2000, we used a paired T-test to compare the eGFR at the start of the therapy between the patients who developed CKD and those who did not. The cumulative incidence of CKD was described using a Kaplan–Meier curve. A binary logistic regression model was used with chronic kidney disease as the dependent variable. The model included gender, age at initiation of lithium therapy and duration of lithium exposure. SPSS^®^ Statistics 18 was used for statistical analysis. p-values < 0.05 were considered statistically significant.

## Results

1012 patients treated with lithium were identified for analysis from the database. Of the study population 55% was female. Mean age at onset of lithium therapy was 44.4 years (± 13.4). The average duration of lithium therapy was 9.2 years (± 8.4). In 118 patients eGFR declined below 60 ml/min/1.73 m^2^ after the year 2000, this comprised 12% of the study population. In this subgroup 60% was female, the mean age at the start of lithium therapy was 48 years (± 12) and the average duration of lithium therapy was 12.4 years (± 8.4). The incidence of an eGFR decline below 60 ml/min was 0.012 per exposed patient year. Incidence for the occurrence of chronic kidney disease stage 4 (eGFR < 30 ml/min) was only 0.0004 per patient year. No cases of end stage renal disease (eGFR < 15 ml/min) were documented in this cohort.

**Fig. 1:**
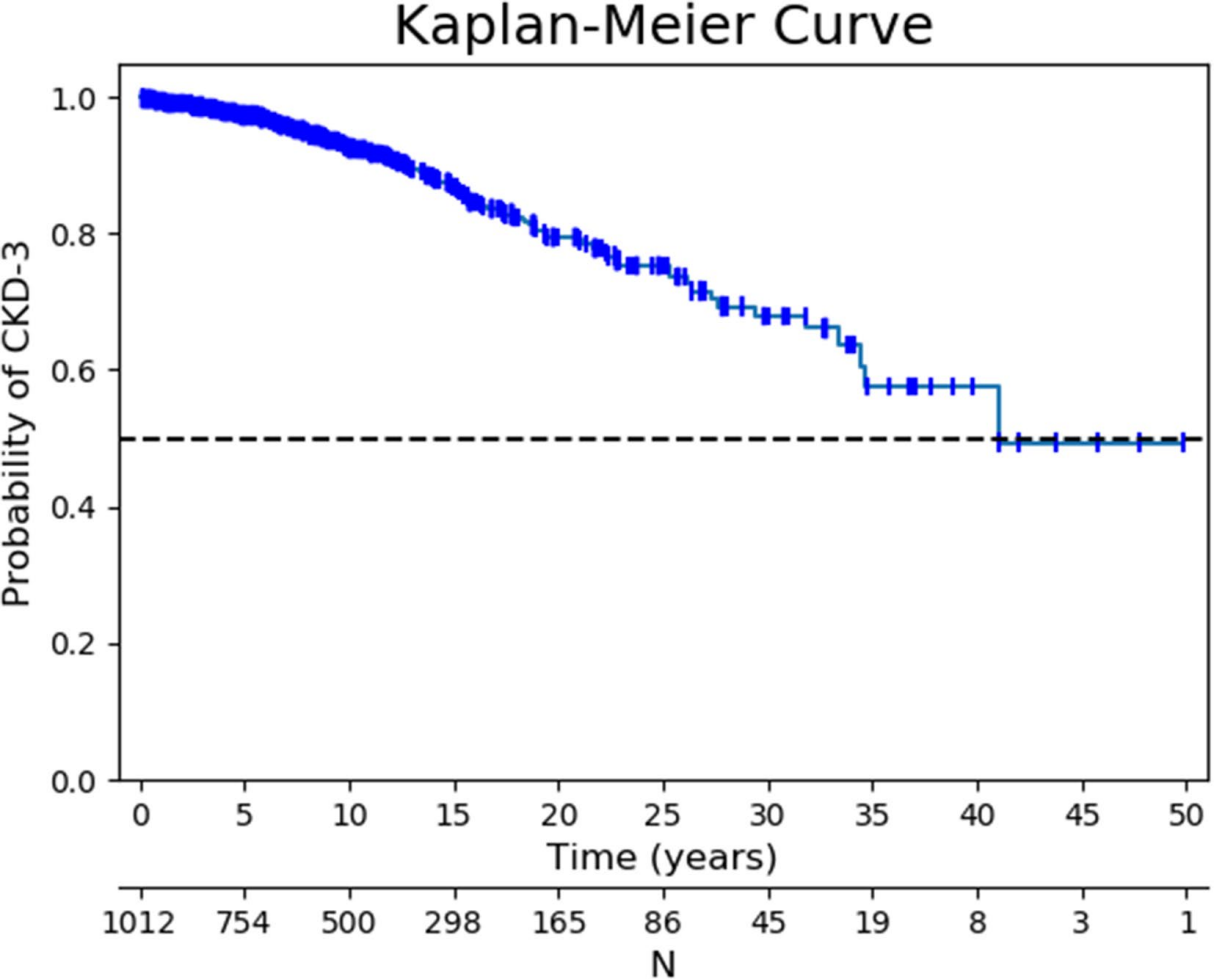
Kaplan Meier curve of CKD-free survival in lithium treated patients

**Fig. 2:**
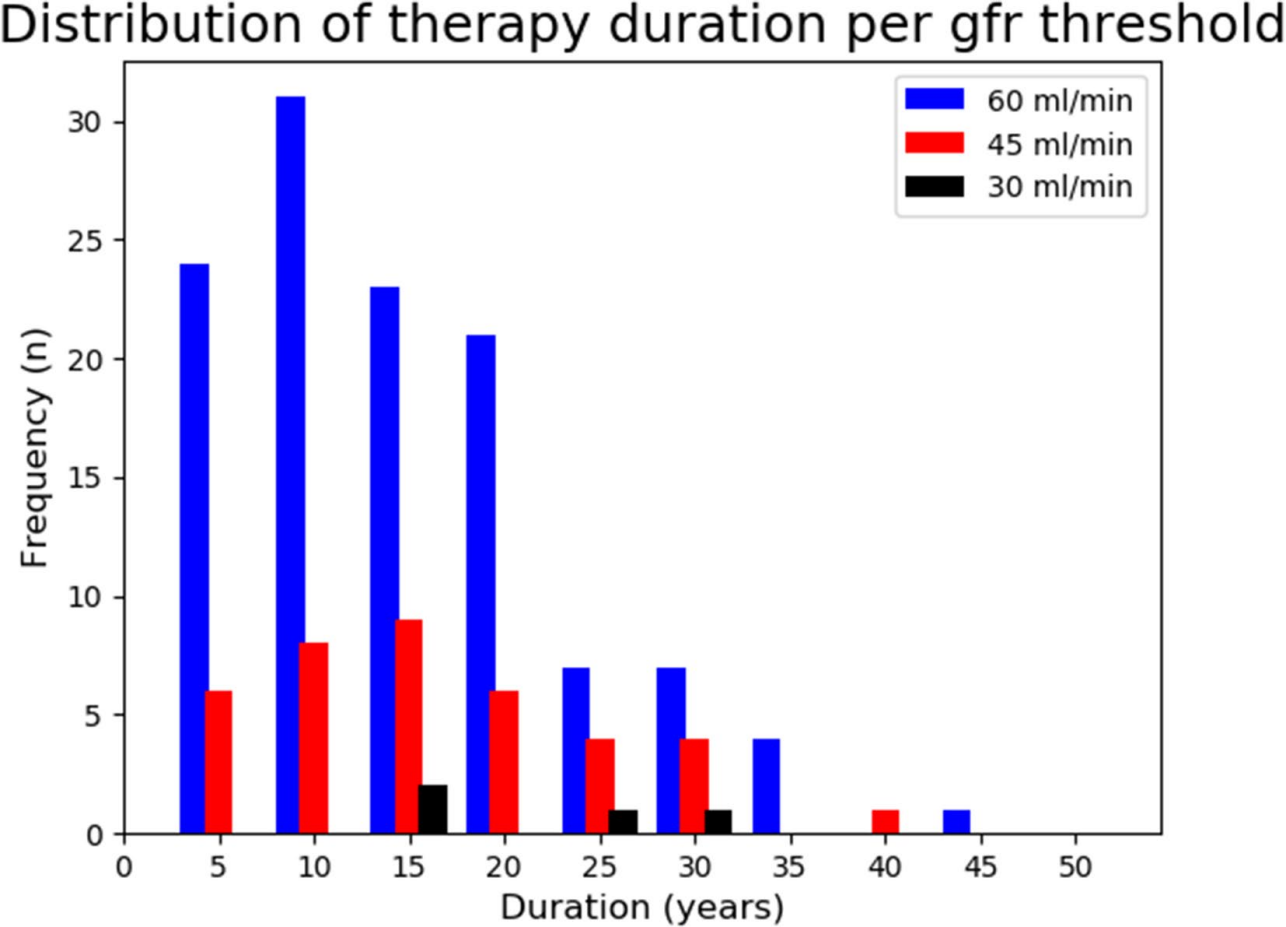
Distribution of therapy duration per GFR threshold

On average eGFR declined 1.8 ml/min/year in the 118 patients who met our target for chronic kidney disease. In the remaining 894 patients eGFR declined only 0.5 ml/min/year. A Kaplan–Meier analysis estimated median time to reaching CKD stage 3 at 40.9 years as shown in Fig. 1. Approximately 10% of patients met our criterion for chronic kidney disease within 5 years after initiation of lithium therapy. A total of 224 patients (13.2%) used lithium more than 20 years without developing CKD. Figure 2 presents the distribution of patients at different CKD stages by the years of lithium exposure.

In the subgroup of patients who started with lithium therapy after 2000, patients who developed CKD had a mean eGFR of 69.8 ml/min at the start of treatment. Patients who did not show a decline of eGFR below 60 ml/min (± 12.2) had a mean eGFR of 80.0 ml/min (± 15.7) at the start of treatment. This difference was statistically significant with a p-value of 0.004.

Age at the start of lithium therapy (odds ratio 1.03 (C.I. 1.007–1.06, p = 0.014)), and duration of lithium exposure (odds ratio 1.03, C.I. 1.01–1.05, p = 0.003) were associated with a decline in renal function. Mean lithium levels were about the same in patients who developed CKD in different stages (e.g. stage 3: n = 118; mean lithium level 0.71 mmol/l, SD 0.12) and those who did not (n = 894; mean lithium level 0.70 mmol/l, SD 0.11).

## Discussion

We performed a naturalistic retrospective observational cohort study of the incidence of chronic kidney disease in lithium-treated patients, to address a major concern of patients and clinicians about long-term lithium treatment. Our results suggest that the incidence of chronic kidney disease, defined as an eGFR below 60 ml/min, in patients treated with lithium is 0.012 per patient-year. The incidence in our population is therefore 1.3 times higher than that of the general population in the Netherlands, where an incidence of CKD stage 3 or more of 0.0093 per person-year was found in a group with a comparable age as our patients (van Blijderveen et al. 2014). This difference is probably explained by ours being a highly selected population of psychiatric patients. In our study population lithium use is an obvious risk factor for CKD but from the current data we cannot conclude it is the sole explanation for an increased incidence rate.

In total 12% of patients reached CKD stage 3 which is comparable to previous studies (Aiff et al. 2015; Tondo et al. 2017). Patients with CKD stage 3 had a mean decline of renal function of 1.8 ml/min/year which is slightly lower than most previous reports (Presne et al. 2003; Tondo et al. 2017). Decline in renal function appears to be most pronounced in the initial years after the start of lithium therapy.

The occurrence of chronic kidney disease stage 4 is rare and end-stage renal disease did not occur at all in our cohort. One possible explanation might be discontinuation of lithium therapy as eGFR approaches 40 ml/min lithium, following recommendations of current clinical guidelines. When lithium is discontinued, follow up of renal function is sometimes transferred to the nephrologist, most probably in the hospital in the same region using the same laboratory, but in an individual case outside our region, making creatinine values unavailable to our database.

Duration of lithium exposure and age at onset of lithium therapy, were found to increase odds of developing CKD stage 3. The same risk factors were found in some other studies (Bocchetta et al. 2013; Davis et al. 2018). From our data longer duration of lithium exposure seem to predispose to decline in renal function. Our analysis suggests, however, that its contribution is relatively low with an increased odds of only 1.03 per exposed patient year.

In clinical practice the treating psychiatrist monitors lithium levels frequently and if necessary adjust the dose to keep those levels in the therapeutic range. Therefore, although mean lithium levels were the same in all groups, we think we cannot conclude that fluctuations in lithium levels don’t contribute to an increased risk for developing CKD.

As suggested by Nielsen et al. (2018) we think closely monitoring lithium levels and renal functioning can largely prevent the occurrence of chronic kidney disease stage 4 and end stage renal failure. If done so, our analysis suggests that long term use of lithium may be considered only an additional risk factor for the occurrence of chronic kidney disease. Rather than focussing on lithium as the main cause of renal failure, monitoring and management of hypertension and diabetes mellitus, as well as other risk factors associated with cardiovascular morbidity, should receive more attention in clinical practice. This may improve renal survival in this category of patients and hence allow them to continue their lithium treatment for a longer period of time, maintaining stability of bipolar disorder and associated better quality of life.

## Conclusions

When carefully monitored the use of lithium seem to cause chronic kidney disease only in a minority of patients. Severe and end-stage renal failure probably occurs seldom.

## Data Availability

The datasets used and analysed during the current study are available from the corresponding author on reasonable request.

## Abbreviations

CKD: Chronic kidney disease
CKD-EPI: Chronic kidney disease epidemiology collaboration
eGFR: Estimated glomerular filtration rate
ESRD: End-stage renal disease
